# Optimizing international trade: Strategies for retaining or taking over transport remit in Poland’s import and export landscape—A case study of Company X

**DOI:** 10.1371/journal.pone.0332126

**Published:** 2025-09-09

**Authors:** Joanna Bednarz, Monika Grottel, Anna Sperska, Giuseppe T. Cirella, Michael Suchanek, Aneta Oniszczuk-Jastrząbek, Ernest Czermanski

**Affiliations:** 1 Faculty of Economics, University of Gdansk, Sopot, Poland; 2 University Center for Social and Urban Research, University of Pittsburgh, Pittsburgh Pennsylvania, United States of America; University of Florida, UNITED STATES OF AMERICA

## Abstract

This study explores the economic implications of transport remit management in Poland’s international trade landscape, with a particular focus on the operations of a medium-sized Polish forwarding company (Company X). Employing a mixed-methods approach, the research combines quantitative analysis of government datasets, firm-level transaction data, and qualitative insights from a targeted industry survey. The case study of Company X reveals notable reluctance among Polish enterprises to assume transport remit responsibilities, particularly in import operations, due to preferences for foreign partners, limited experience with international logistics, and concerns about administrative complexity. Analysis of Incoterms® usage patterns highlights a partial recovery of forwarding services in 2021, reflecting post-pandemic adjustments in logistics strategy. While the findings cannot be generalized to the entire Polish economy, they offer a detailed, data-driven illustration of the microeconomic factors influencing transport remit decisions. The study underscores the need for transparent procedures, legal clarity, and targeted training initiatives to increase the uptake of transport remit services by domestic firms. By improving internal capabilities and reducing reliance on foreign partners, Poland could strengthen its position in global trade and enhance value creation in the logistics sector.

## 1. Introduction

Transport remit refers to the agreed terms and responsibilities between parties in a commercial transaction—typically importers and exporters—regarding the movement of goods. Central to this is the selection of *Incoterms®* (International Commercial Terms), standardized rules issued by the International Chamber of Commerce (ICC) that clearly define which party is responsible for transportation, insurance, customs clearance, and related costs [1]. By establishing shared expectations, Incoterms® reduce legal disputes and streamline international and domestic trade processes.

Effectively managing transport remit allows enterprises to optimize internal transport resources, supporting more strategic and cost-effective logistics operations [2,3]. This can significantly influence the structure and scale of transactional handling costs, ultimately affecting cargo pricing [4]. Moreover, transport remit decisions can offer considerable opportunities for additional revenue or cost savings, sometimes matching the importance of a firm’s core business activities [5,6]. In broader terms, transport remit management can also contribute to national strategies aimed at expanding international trade.

In Poland, a notable share of trade involves interactions with foreign entities, incorporating both imports and exports. This active international engagement holds the potential to influence the negotiation and establishment of terms related to transportation in these operations. Research from Knap [7] and Urbanyi-Popiołek [8] reveal that in certain regions of the country, numerous enterprises forego transport management in imports, opting for specific Incoterms® trade formulas like Carriage Paid To (CPT), Carriage and Insurance Paid To (CIP), Cost, Insurance, and Freight (CIF), and Delivered At Terminal (DAT). Similarly, in terms of exports, a substantial number of enterprises relinquish management control of transport by employing formulas such as Ex Works (EXW), Free Carrier (FCA), and Free On Board (FOB). Interestingly, the recent findings of Hajdukiewicz and Pera [9] indicate that preferred import transactions are influenced by dominant players with significant bargaining power and industry-specific considerations, utilizing formulas like Delivered At Place (DAP), FOB, and CIF. Meanwhile, the most common export formula remains EXW, followed by DAP, CIF, and FCA. These discrepancies over that past eight or nine years have been observed across different transport modes and regions of the country, underscoring the importance of researching Incoterms® practice.

Existing empirical studies in Poland are constrained to regions with proximity to seaports, potentially biasing Incoterms® formula selection. In addition, industry and geographical distinctions are often overlooked. As a result, this study seeks to comprehensively explore the extent and structure of transport remit adoption in Poland’s foreign trade, particularly in areas where it is absent or being neglected. The study aims to uncover motives behind adoption or abandonment, differentiate between adopters and non-adopters, evaluate the benefits for enterprises, identify determining factors for specific benefits, and assess the role of transport remit in contributing to the national economy’s added value. This knowledge is indispensable for importers, exporters, and logistics operators supporting their activities, facilitating a nuanced understanding of transport remit as an effective means for enhancing transport operations and financial gains in foreign trade transactions. Furthermore, this dynamic has the potential to transform the relationships between logistics operators and foreign trade enterprises, fostering mutually advantageous financial partnerships.

To guide this analysis, the following research questions are posed:

RQ1: What are the economic and strategic implications of transport remit management in Poland’s international trade?
RQ2: What factors contribute to the reluctance of Polish enterprises to assume transport remit responsibilities?
RQ3: How do Incoterms® usage patterns affect transaction margins and overall trade competitiveness for Polish forwarding firms?

The research is structured into three interconnected parts. It begins with a comprehensive examination of both theoretical and empirical studies on Incoterms® and transport remit. Following this, the research methodology is outlined, providing a clear framework for the study. Finally, the research concludes with a discussion of the empirical findings and offers recommendations for future research and potential improvements in business practices.

## 2. Literature review

Import and export play pivotal roles in all industries, where trade and production costs significantly impact competitiveness on the global stage [10]. As highlighted by Chamsuk [11], the negotiation of trade agreements is intricate and diverse. Therefore, an importer-exporter trade agreement or adherence to international terms of supply is paramount, as it delineates roles, responsibilities, transport liability, and compensation universally recognized [12]. Incoterms® serve as the standardized terms and conditions for delivering goods, constituting the international benchmark for sales contracts between buyers and sellers. These terms have been codified by the ICC and align with United Nations trade rules. The ICC [13] first published Incoterms® in 1936, featuring six terms and principles: (1) Free Alongside Ship (FAS), (2) Free Alongside Ship-Free On Board (FAS-FOB), (3) Cost and Freight (CFR), (4) CIF, (5) Ex Ship, and (6) Ex Quay. The ICC updates Incoterms® every decade, with the previous edition released in 2010 [14–17].

The latest release, Incoterms® 2020, came into effect on January 1, 2020 [1]. Notable distinctions between Incoterms® 2010 and Incoterms® 2020 encompass: the conversion of DAT to Delivered at Place Unloaded (DPU); clarifications regarding insurance points in CIF and CIP; improved transparency concerning costs and cost structures; detailed specifications outlining transport safeguards; provision allowing the use of proprietary transport instead of relying solely on third-party transport; clarifications on FCA, FOB, and waybills; and enhanced user-friendly presentation and design [18]. The selection of Incoterms® formula in sales contracts is influenced by a myriad of factors. These include the nature of the product and shipment, the characteristics of the trader, and their market power, which can be categorized as equal, dominant, or subordinate. Additionally, the complexities of the supply chain and broader macroeconomic and microeconomic considerations play pivotal roles in shaping this decision-making process.

Drawing from a comprehensive literature review, Hien *et al*. [19] pinpointed key factors influencing the selection of Incoterms® formulas. Their analysis underscored destination country risk as the paramount factor, with company size, resources, negotiating power, competitive advantage, regulatory measures in the target market, product attributes, and the international experience of the enterprise also making significant contributions. Subsequently, through their own survey, they ascertained that the frequency of employing Incoterms® formulas, coupled with a deep understanding of their significance, has a measurable impact on export performance. This influence extends to financial outcomes, strategic effectiveness, and managerial satisfaction with export activities. The study highlighted that firms possessing knowledge of Incoterms® formulas exhibit a heightened ability to discern environmental factors, leading to enhanced export performance. Additionally, the research established a correlation between export performance and a company’s negotiating prowess and competitive advantage in foreign markets.

Similarly, Unal and Metin [20] investigated the factors that influence the choice of Incoterms® formulas in both import and export scenarios. Their findings revealed that three overarching factors—transportation costs, cost of goods, and payment methods—resonate as equally crucial for both scenarios. Subsequently, other shared considerations included risk and custom processes (i.e., bureaucracy), indicating common concerns across both sides of the trade transaction [21]. However, divergent perspectives emerged on factors like relationships with forwarders, types of goods, transportation complexity, and distance [22]. Importers, notably, place a greater emphasis on the nature of goods, whereas exporters are more influenced by distance.

Additionally, extensive research has delved into the determinants influencing the selection of Incoterms®, particularly regarding their impact on export efficiency [23–26] and their role in negotiation and communication in logistics decision-making between sellers and buyers [27]. Specifically, Suraraksa *et al*. [28] conducted a study examining the factors driving decision-making regarding Incoterms® among auto parts manufacturers in Thailand. Their investigation identified criteria such as operating costs, cooperation and bargaining power, knowledge and understanding, and the duration of operations. The study concluded that FCA emerges as the most suitable Incoterms® formula for international trade among auto parts manufacturers. One reason for this preference is that FCA allows car manufacturers to retain control over the delivery of vehicle parts to the carrier, ensuring adherence to quality standards and specifications. Additionally, by maintaining control until the vehicle parts are handed over to the carrier, manufacturers can mitigate the risk of damage or loss during the initial transportation stages.

Exploring the dynamic landscape of digital logistics and supply chain management, Durdağ and Delipinar [29] put forth a compelling case for the forthcoming evolution of Incoterms® formulas, which are intricately tied to considerations of location, temporal constraints, and financial arrangements. While they foresee minimal adjustments in the forthcoming Incoterms® 2030 update, they anticipate substantial revisions in both the scope and substance of terms set to debut in 2040. Consequently, the concept of transport remit, as expounded by Neider [30], not only delineates the rights, benefits, risks, and obligations of trading partners concerning transport management (and associated cost considerations) but also represents a collaborative decision made during contract negotiations, with the potential for mutual sharing between contracting parties. As such, the benefit of having a transport remit lies in its capacity to shape the entire transport chain, as highlighted by Kotowska and Letmanski [31]. This operation wields a direct influence over various facets of the commercial transaction and the transport process, including customs clearance, determination of delivery volume and frequency, selection of transport routes, designation of loading and unloading points, choice of transport mode and type, subcontractor selection (e.g., forwarder, shipowner, and carrier), delivery timelines, cargo insurance, and document circulation. Such an enterprise is strategically positioned to secure additional financial advantages by leveraging opportunities for increased profit or realizing savings, particularly in response to fluctuations in freight prices. This is particularly evident when transport can be managed at a lower cost than initially factored into the price of the goods.

One notable advantage lies in the capacity to utilize one’s own transportation assets or negotiate favorable transport terms with established partners, i.e., usually facilitated through long-term collaboration [32]. Extensive research on international trade costs delves into multifaceted dimensions, from the intricate interplay between transport costs and trade structures [33] to the strategic adoption of Incoterms® formulas [34], and broader trade considerations [35,36]. However, assuming the role of transport remit entails significant responsibilities, necessitating detailed logistics planning and financial support. As a result, transport remit carries inherent risks, including challenges in securing specific services, efficiently coordinating transportation, and navigating price fluctuations in freight. Furthermore, the potential for non-compliance with contractual terms and delays in the transport process adds complexity [8,37].

Moreover, assessing the feasibility of running transport remit services necessitates a comprehensive evaluation of both commercial and non-commercial risks. Commercial risks encompass uncertainties in transactions, such as partner insolvency, document errors, and cargo damage or loss during transit, as well as risks associated with specific transportation modes. Conversely, non-commercial risks stem from evolving environmental factors, including natural disasters and political instability [8,32,38,39]. Additionally, exporters operating under international supply contracts face the risk of non-payment, as emphasized by Bergami [40]. As such, strategically employing international supply terms can serve as a vital tool in mitigating payment risks. Dinçer and Karakuş [41] identified exchange rate, political, and payment risk as pivotal factors in this process. Petrusheva [42] underscores the risk of payment delays, particularly when conducting sales on credit, a common practice in international trade. In addition, Kramarz *et al*. [43] draw attention to the risk of volatility in trade flows, as exporters often heavily depend on a limited number of major customers, leaving them susceptible to microeconomic demand shocks. Hence, it can be stated that the spectrum of risks in international trade encompasses legal, financial, and operational dimensions, prompting companies to implement robust risk management strategies to navigate potential volatility [44].

According to Urbanyi-Popiolek [8], small and medium-sized enterprises (SMEs) often refrain from using transport remit services due to concerns about incurring transportation costs. This reluctance may stem from a lack of relevant experience, shortage of qualified employees equipped with the necessary knowledge and negotiation skills for delivery terms, unfamiliarity with the impact of assuming transport remit services on the goods’ pricing, a hesitancy to organize deliveries, and a general unfamiliarity with the forwarding and transport market. Additionally, SMEs often lack their own legal departments and do not engage law firms for support (which may incur additional legal fees). Moreover, the primary barriers to exporting vary across different contexts. In the case of Indonesian SMEs, research by Revindo [45] revealed that the types and severity of export barriers differ across export stages and industries. In China, intra-national barriers arising from local protectionism pose a significant challenge for both exporting SMEs and those looking to export [46]. Meanwhile, in the Western Balkan region, non-tariff barriers, particularly those stemming from political and ethnic disputes, significantly impede intra-regional SME trade [47]. Additionally, incomplete value added tax (VAT) refunds can function as a de facto tax on exporters’ input purchases, reducing SME exports in developing countries [38,48].

Drawing insights from a survey in Poland involving 709 exporters and non-exporters, Gawlikowska-Hueckel and Umiński [49] present a hierarchy of export barriers, ranked from the most to the least significant. These barriers encompass heightened competition on foreign markets, exposure to exchange rate risks, insufficient support for exports, limited access to information about business opportunities in foreign markets, the non-introduction of the euro currency in Poland, inadequate demand in foreign markets, difficulties in collaborating with other companies to enter foreign markets, and a lack of foreign language proficiency among company personnel. Aligning with these findings, experts from the Center for the Development of Small and Medium-sized Enterprises in Poland, conducted another survey among Polish exporters in 2012, identifying key barriers [50]. Exchange rate risk emerged as the predominant concern, followed by limited access to reports and analyses, weak recognition of Poland’s brand, and challenges in financing exports through internal funds. From a Polish standpoint, exporters face various challenges when venturing into international markets, including heightened competition, bureaucratic complexities, elevated risks stemming from unfamiliar markets, cultural barriers, intricate legal matters, limited demand for Polish goods, and issues related to corruption [9,51].

Surprisingly, within Poland, transportation and warehousing costs have emerged as the most influential factors in the selection of Incoterms® formulas. By contrast, aspects such as relationships with counterparties, payment methods, and modes of transportation play a less decisive role. Additionally, internal enterprise variables—such as staff expertise, access to financial or physical resources, and the overall value of the transaction—are generally of minor importance in this decision-making process [9]. Within this framework, the Authorized Economic Operators (AEO) program, introduced by the European Union (EU) in 2009, holds particular relevance. The program, which grants customs simplifications and security-related benefits to compliant EU-based importers and exporters, serves as a strategic tool for enterprises seeking to assume greater control over transport remit services. Research on AEO-certified firms across EU Member States provides key insights into operational best practices and offers a basis for tailored recommendations to encourage broader adoption of transport remit by Polish enterprises, both in domestic and international trade [52,53].

## 3. Materials and methods

### 3.1. Data collection

The quantitative and qualitative research drew data from three distinct sources: government datasets, a medium-sized Polish forwarding company case study, and an industry-wide survey. First, to analyze the share of individual Incoterms® in foreign trade transactions conducted by Polish enterprises, investigating the potential to assume transport remit where it is not traditionally used, the study utilized databases from the Department of the Analytical Center of the Chamber of Tax Administration in Warsaw (*Wydział Centrum Analityczne Izby Administracji Skarbowej*), Statistics Poland, and the Tax Office. These comprehensive datasets aided in piecing together the actual commodity trade transactions between January 1, 2019, and December 31, 2019, as well as between January 1, 2021, and December 31, 2021 [54]. The data, declared in customs declarations, i.e., single administrative documents, and Intrastat statistical declarations, includes information on imports and exports of goods categorized by country of origin and country of consignment [46,54]. Encompassing 79 Harmonized System (HS) divisions, excluding four sections [50], the datasets include further details on net mass, statistical monetary value, customs procedure code, mode of transport code, delivery terms code, and the number of entities that made customs declarations for a specific customs procedure. The determination of the number of entities was collective for each HS division and the Konrad-Adenauer-Stiftung Analysis Center provided and analyzed the data, segmented into two categories: import and export.

Second, concerning the foregone benefits, the adverse repercussions of Polish importers and exporters relinquishing transport remit to foreign partners impact four key stakeholders: seaports in Poland, forwarding enterprises, the State Treasury, and the importers and exporters themselves. To ascertain the extent of these lost benefits, primary data were sourced from a leading Polish forwarding company, hereafter referred to as “Company X,” specializing in facilitating shipments for domestic importers and exporters globally, with a primary emphasis on sea freight. The case research delved into an exhaustive list of all sea trade operations conducted in 2021 and the first half of 2022, extracted from Company X’s operations. This encompassed details such as transport direction, loading and destination points, Incoterms® formulas, and financial metrics characterizing each transaction, including sales and margins. The dataset covered a total of 29,085 forwarding operations, handling 81,366 twenty-foot equivalent units (TEUs), along with bulk container and bulk cargo operations. The case study enabled the identification of specific nuances within the forwarding services for Polish importers and exporters, shedding light on the average values of margins generated across different stages of the supply chain.

Third, a qualitative component was implemented through an industry-wide survey targeting Polish logistics and forwarding managers. The goal was to identify the key factors driving Polish enterprises to relinquish control of transport remit services to foreign partners. The survey provided valuable insights from logistics professionals, shedding light on the operational and strategic considerations behind these decisions. Data collection occurred between July and October 2022 via an online questionnaire. Invitations were distributed by email to managerial personnel within Poland-based enterprises actively engaged in maritime transport and customs representation. Candidate firms were identified using the 2022 Transport, Shipping, and Logistics (TSL) ranking and the EU’s AEO database, thereby focusing on companies with substantial market presence and recognized compliance status. To ensure data reliability, the survey specifically targeted senior managers and operational staff directly involved in forwarding and customs processes. A total of 32 valid responses were collected, constituting 21.2% of the invited sample. Although the sample size is limited, it represents a strategically selected, knowledgeable cross-section of the maritime logistics sector. All responses were carefully reviewed, and measures were taken to minimize bias by ensuring coverage across diverse enterprise sizes and operational scopes.

### 3.2. Data analysis

To extrapolate the findings, the study utilized four econometric models to depict the profitability of transactions with and without transport remit in both imports and exports. Import transactions with transport remit adhered to Incoterms® formulas from groups E and F, while those without transport remit were regulated by formulas from groups C and D. Conversely, for export transactions, formulas from groups C and D denoted transactions with transport remit, whereas those from groups E and F indicated transactions without transport remit.

The choice to employ a partial equilibrium econometric model—rather than broader models commonly found in the literature, such as computable general equilibrium (CGE) models—was driven by the study’s microeconomic focus. The partial equilibrium approach provides precise, transaction-level insights into the economic effects of transport remit practices, making it especially suitable for analyzing firm-specific data sourced from Company X and customs declarations. This method allows for the direct estimation of margins and the financial impact of Incoterms® usage on operational profitability. By contrast, CGE models are typically applied to assess economy-wide interactions across multiple sectors, offering macro-level simulations. Since this study aims to identify value losses at the firm and sector level, the partial equilibrium framework is not only more appropriate but also consistent with modern analytical practices in logistics and international trade research [33,41].

To achieve this, the overall transaction structure was compared to ensure its representativeness for the Polish economy, with margin figures exhibiting positive deviations in relation to the average values of commercial economic parameters. Discrepancies were also observed in the structure of containerized transactions, necessitating the application of appropriate adjustment parameters for both aspects. Separate models were developed for imports and exports, where the explanatory variables included the margin per operation and the margin per operation per 1 kilogram, respectively. The Incoterms® groupings (i.e., C/D and E/F) served as the explanatory variable, while control variables encompassed distance (i.e., port-to-port), cargo weight, and the month of the transaction. The estimation process was carried out using TIBCO Statistica Version 13 software, assuming a normal distribution and employing logarithm as the link function.

The empirical data underwent a comprehensive statistical analysis. Initially, descriptive statistics were employed to categorize the data into quartiles based on their distribution. Fit tests for normal distribution, including the chi-square test and the Kolmogorov-Smirnov continuous test with Lilliefors corrections, were conducted. Recognizing that Incoterms® groupings did not follow a normal distribution, a non-parametric statistical toolkit was applied. To ensure the development of a robust and reliable model, numerous correlation and comparison analyses were conducted. Spearman’s correlation coefficient was calculated to assess the strength of the correlation between Logistics Performance Index values and the Incoterms® groupings, as well as between weight and value shares within the same group. Finally, a regression model was constructed, utilizing the presented input data, to predict economic effects accurately.

In analyzing the performance of Company X, renowned for its extensive experience and strong competitive position in the Polish market, an evaluation of its transport transactions was conducted. With a comprehensive service offering spanning sea, land, air, rail, and intermodal transport, notably in sea forwarding, the focus was on assessing the balance between transactions with and without transport remit in both imports and exports. This assessment aimed to validate the trend of Polish enterprises relinquishing transport remit to foreign partners. Additionally, the study involved quantifying the margin losses incurred by Company X due to transactions conducted without transport remit. Furthermore, a comparative analysis was conducted for the years 2019 and 2021 to juxtapose Company X’s utilization of transport remit services, assessing whether it had reverted to pre-pandemic levels. This analysis provides insights into the potential differences and the future trajectory of Poland’s TSL industry. Finally, the study sought to estimate the economic impact on the Polish economy arising from the growing trend of transport remit services to foreign partners by Polish importers and exporters.

### 3.3. Ethics statement

This study was conducted in accordance with the ethical guidelines and regulations of the University of Gdansk. The research protocol, including the survey of logistics and forwarding managers, was reviewed and approved by the Ethical Committee of the University of Gdansk. The study complies with the principles outlined in the Declaration of Helsinki and adheres to applicable ethical standards and legislation in the European Union.

Participation in the survey was voluntary, and all participants were informed about the purpose of the study, the confidentiality of their responses, and their right to withdraw at any time without consequence. No personally identifiable information was collected, and all responses were anonymized prior to analysis. Consent was obtained electronically from all respondents before participation in the survey.

## 4. Results and discussion

### 4.1. Macroeconomic significance and sectoral absorption

The economic significance of transport remit services in Poland extends far beyond freight forwarding. These services contribute meaningfully to national value added and the overall efficiency of supply chains. In 2021, the transport and storage sector represented approximately 6.3% of Poland’s gross value added—equivalent to PLN 172.6 billion (USD 44.2 billion)—and supported 7.3% of total employment [55]. Within this sector, freight forwarding and logistics play an important role in shaping the cost-efficiency and reliability of foreign trade operations, especially through strategic management of transport remit [38,52].

To complement the analysis of the sector’s macroeconomic weight, it is equally important to examine how the provision of transport remit services is absorbed by the broader economy. The transport and logistics sector serves as a foundational enabler for economic activity, particularly in an export-oriented economy like Poland’s. Transport remit services—when assumed by domestic enterprises—generate downstream demand across multiple sectors including warehousing, port services, fuel and energy, insurance, and customs brokerage. This cross-sectoral interaction illustrates the sector’s multiplier effect. According to data from Statistics Poland [56] and the Polish Economic Institute [57], expenditures related to logistics services have an estimated multiplier of 1.6 to 1.8 in terms of contribution to GDP, meaning that for every PLN 1 (USD 0.26) invested in transport and logistics, the economy generates PLN 1.60 (USD 0.42) to PLN 1.80 (USD 0.47) in added value.

Moreover, the absorption of these services reflects structural readiness—i.e., the availability of skilled labor, modern infrastructure, and legal and administrative facilitation. Poland’s ongoing investments in intermodal hubs and EU-supported transport corridors further enable the integration of transport remit services into international supply chains [9]. This is especially critical for industries adopting Just-In-Time (JIT) production models, where even slight disruptions in logistics can halt entire manufacturing processes. While JIT is not an Incoterm®, it underscores the importance of reliable, precisely timed deliveries—making domestic transport remit capabilities even more strategically valuable [2,3,6].

In practice, the main demanders of transport remit services in Poland are logistically intensive and globally integrated industries characterized by high-volume exports, complex supply chains, and dependency on timely delivery—namely, the automotive sector, electronics, advanced machinery, and the food processing industry [2,38,49]. These sectors depend on high-frequency, cost-sensitive shipments where control over transport terms can directly affect pricing, customer satisfaction, and competitiveness [4,9]. Despite this potential, as the findings of this study show, many Polish firms defer to foreign partners due to perceived advantages and a lack of awareness or capacity to manage transport logistics [52]. Enhancing the domestic supply of these services—through upskilling programs, infrastructure incentives, and simplified regulations—could improve absorption capacity and strengthen Poland’s position in global trade networks. Ultimately, the provision and absorption of transport remit services play an important role in shaping national competitiveness [31,53].

### 4.2. Unlocking potential: Insights from Polish forwarding enterprises

Drawing from comprehensive datasets from Poland, several notable insights emerge regarding the role of transport remit in foreign trade. Each year, approximately 31.7 million tonnes of exports—about 43% of Poland’s total annual export volume—are not managed by Polish forwarding enterprises, representing a significant unrealized domestic opportunity. The value of these exports reaches 52% of Poland’s annual export value, totaling around USD 104 billion, highlighting the economic weight of this unclaimed segment [54,55].

Further analysis indicates that in import transactions, 56% of operations occur without Polish involvement in transport remit services. For exports, around 48% of transaction volume similarly bypasses domestic transport remit management segment [54]. This means a substantial portion of foreign trade logistics—and the associated value-added—is effectively ceded to foreign entities. This underutilization persists despite the presence of domestic capabilities and infrastructure, including expanding intermodal hubs and EU-supported logistics corridors. If addressed strategically, these gaps could provide a pathway to enhance Poland’s share of logistics value creation, employment, and global competitiveness in foreign trade.

Within the domestic forwarding market, transport remit services are more frequently observed in operations conducted with foreign partners. However, these practices are comparatively underdeveloped domestically, underscoring the importance of better integrating Polish enterprises into transport decision-making processes in foreign trade transactions.

Building on this, the case study of Company X reveals a clear pattern: a significant share of both import and export transactions—39% and 33%, respectively—are handled by foreign partners, effectively bypassing Polish forwarding enterprises. This trend reflects a broader pattern in Poland’s international trade landscape, emphasizing an increasing reliance on foreign partners for transportation responsibilities, as evidenced by prior research [8,9,53]. Crucially, orders with transport remit consistently yield higher margins compared to those without, indicating the potential financial benefits of retaining control over transportation logistics. Dominant Incoterms® formulas further illustrate this preference, with FOB and CIF comprising 85% of handled orders in imports, while CFR, CIF, DAP, and FOB dominate exports, accounting for 82% of orders. Despite the margin loss observed in 2021 due to the COVID-19 pandemic, attributed to the unique dynamics of the maritime container freight market during that year, a broader analysis may reveal a lower margin loss in more typical years. This highlights the importance of evaluating long-term trends and industry dynamics for informed decision-making in transport remit strategies.

Additionally, the nuanced dynamics within the Polish logistics landscape are evident in regional disparities in transportation management. For instance, western Polish ports exhibit a stronger inclination towards selecting transport remit services with a domestic partner, highlighting the distinctive approaches adopted in different regions. While expecting Polish enterprises to assume complete control of transport remit might be unrealistic, estimating the value of lost margin acts as a catalyst for discussions on the potential expansion of transport remit within the Polish economy. These conversations could provide valuable insights into boosting Poland’s competitiveness in global trade by refining transport management strategies and promoting collaboration among domestic stakeholders in the logistics sector.

### 4.3. Added value computing

The statistical analysis of the TSL of Company X’s data revealed that, across all transactions, 64% of forwarding operations involved transport remit by Polish partners, while 36% featured foreign partners. When distinguishing between import and export transactions, a comparable pattern was observed, suggesting a slightly greater inclination to utilize transport remit services in exports than in imports (Table 1). In import transactions, FOB, CIF, and EXW formulas were predominant, constituting 90% of total transactions. Conversely, among export transactions, CFR, FOB, and CIF formulas played a prominent role, comprising 69% of total transactions.

**Table 1:** Distribution of operations with and without transport remit in Company X versus the domestic Polish market, segmented by import and export transactions in 2021.

Operation	Company X		Poland	
	Import	Export	Import	Export
Transactions with transport remit by Polish partners	61%	67%	24%	42%
Transactions with transport remit by foreign partners	39%	33%	56%	32%
Transactions without an Incoterms® formula	—	—	20%	26%

In the context of full container load (FCL) imports, a notable divergence from the norm was observed regarding the utilization of transport remit. Nearly equal proportions were observed between operations with and without transport remit, with 46% opting not to use it and 54% choosing to utilize it. This trend extended to the container count (i.e., TEUs) handled. Conversely, FCL export operations displayed a strong preference for transport remit from Polish exporters, constituting a significant 66% of transactions and rising to 71% when measured by TEUs.

In contrast, bulk goods forwarding leaned heavily towards transactions without Polish transport remit. Notably, while bulk goods make up a small fraction of Company X’s revenues, import transactions dominate the directional flow of forwarding operations at 90%. This dominance is echoed in tonnage handled, indicating that orders managed with Polish partners tend to be larger.

Analyzing containerized general cargo, specifically less than container load (LCL), revealed a significant majority of transactions utilized transport remit services, accounting for 83% of forwarding operations. Import transactions substantially outnumbered exports, making up 82% of transactions with transport remit, while exports comprised 86%. Owing to the considerable weight variation in general cargo shipments and incomplete records in the operating system, a weight-based structural analysis was not feasible.

#### 4.3.1. Analyzing margin disparities: Insights into transport remit impact on Company X’s operations.

The principal objective of the case research was to collect data for extrapolating the lost benefits across the economy. This extrapolation relied on highlighting the disparities in margins earned by the freight forwarder between transactions with and without transport remit on behalf of Company X. To achieve this, the average margin was examined using three distinct approaches.

**Fig 1:**
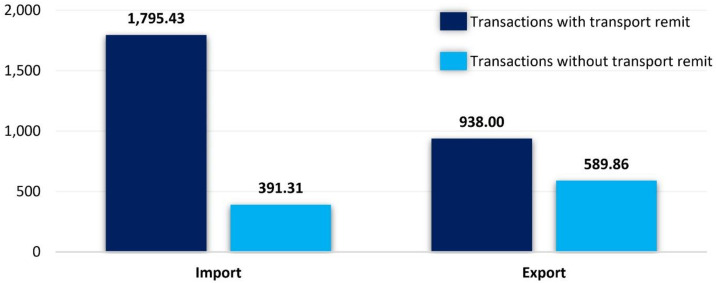
Comparison of average margin gain in total forwarding transactions with and without transport remit by Company X in 2021, USD.

First, the analysis involved assessing the margin per average transaction, both with and without transport remit, considering the overall operations (Fig 1) as well as transactions with transport remit by Polish versus foreign partners for specific transport products (Table 2). The results highlighted a notable disparity in the average shipper’s margin between transactions with and without transport remit. Importantly, transactions involving transport remit by Polish partners consistently yielded a substantially higher margin, evident in both imports and exports. This finding underscores the financial advantage associated with operations where Polish partners takes control of transport remit.

**Table 2:** Average margin gain per forwarding transaction with transport remit for Polish and foreign partners across specific transport products by Company X in 2021, USD.

Transactions with transport remit by Company X	Type of transport products					
	Bulk cargo		LCL		FCL	
	Import	Export	Import	Export	Import	Export
Polish partners	1,863.16	2,142.83	434.02	301.01	2,512.58	986.92
Foreign partners	2,235.89	742.48	185.87	316.65	333.77	597.06

Second, after conducting a comparative assessment of the average margin per operation, considering transactions involving transport remit from both Polish and foreign partners across specific transport products, the analysis was extended to evaluate the amount of margin per 1 TEU handling in FCL transport (Table 3). Across both metrics, transactions involving transport remit with Polish partners consistently demonstrated a significant advantage. The sole exception was observed in the importation of bulk cargo, where transactions with foreign partners outnumbered those with domestic ones.

**Table 3:** Average margin gain per 1 TEU for FCL transport with transport remit for Polish and foreign partners by Company X in 2021, USD.

Transactions with transport remit by Company X	Import	Export
Polish partners	929.10	195.80
Foreign partners	117.87	146.66

Third, the study delved into the monthly fluctuations in margin amounts per forwarding operation, encompassing both transactions with and without transport remit, as observed by Company X. The analysis revealed fluctuations in margin amounts across import and export orders, which remained steady despite the consistently higher margins in transactions utilizing transport remit. These fluctuations can be attributed to various factors, including changes in freight rates, shifts in demand for transport services, and modifications in the commercial activities of Company X.

#### 4.3.2. Balancing margins and costs: evaluating the impact of transport remit on Company X’s profitability.

Overall, the analysis reveals that transactions handled with transport remit yield higher margins for Company X compared to those without. However, to fully evaluate Company X’s profitability, it is crucial to consider how this additional margin offsets the costs associated with increased labor and time demands. Conducting such a study using operational data poses challenges, requiring a prolonged observation period supported by tools measuring time spent on individual orders by traders and forwarders. Additionally, implementing appropriate accounting methods to allocate costs between orders with and without transport remit is necessary for accurate results.

The analysis of Company X underscores the profound influence of transport remit on various facets of its operations, encompassing the structure of transport products and directions, as well as the margin disparity between orders with and without transport remit. This study facilitated the estimation of the forgone value by the forwarder in orders without transport remit across diverse forwarding products (Table 4). It is important to acknowledge that these figures are approximations, given the rarity of transport remit services exclusively residing with Polish partners in every transaction. Additionally, it is vital to contextualize these findings within the distinctive dynamics of the container sea freight market in 2021, i.e., one year into the COVID-19 pandemic. A comparative analysis of more typical years is anticipated to reveal a diminished magnitude of margin loss in transactions without transport remit.

**Table 4:** Estimated margin loss for transactions without transport remit by Polish partners for Company X in 2021, USD.

	Type of transport products								Total
	Bulk cargo			LCL			FCL		
	Import	Export	Import		Export	Import		Export	
Margin loss amount	—	5,602	148,893		854	9,781,529		402,121	10,339,002

### 4.4. Econometric model analysis

The examination of Company X, especially regarding the variations in margins between transactions with and without transport remit, served as the foundation for constructing a comprehensive econometric model. The econometric model sought to elucidate the magnitude of potential revenue losses incurred by the freight forwarding industry in Poland. By analyzing the intricate interplay between different variables, including transaction volumes, profit margins, and market dynamics, the model aimed to provide actionable insights for stakeholders within the logistics sector. Moreover, the utilization of such a robust analytical framing underscored the importance of addressing the systemic challenges associated with transport remit practices in Poland. Through a thorough examination of the data and subsequent modeling efforts, the aim was to pave the way for informed decision-making and strategic interventions aimed at optimizing the efficiency and profitability of freight forwarding operations.

#### 4.4.1. Estimation models for assessing margin loss in import and export operations for Company X.

In analyzing the import and export operations of Company X, numerous models were constructed to assess transaction efficacy, paving the way for strategic decision-making aimed at enhancing operational performance and bolstering profitability. Central to these models is the explanatory variable, which encapsulates the outcome per operation, providing critical insights into the effectiveness and financial ramifications of each individual transaction within Company X’s import and export portfolio. These models were specifically tailored to estimate the margin loss associated with import (Table 5) and export (Table 6) transactions involving the use of transport remit services as well as the outcome per 1 kilogram per operation. Parameter estimations were conducted using a normal distribution, while the link function employed a logarithmic function to capture the nuanced relationships within the data. The findings of this analysis, encompassing estimate values, standard errors, Wald statistics, and confidence intervals at 95%, along with *p*-values, offer insights into the dynamics of import and export transaction performance for Company X and the broader context of Poland’s seaport operations.

**Table 5:** Estimation model for margin loss in import operations for Company X in 2021.

Effect	Parameter estimates Distribution: NORMAL Link function: LOG							
	Level of effect	Column	Estimate	Standard error	Wald stat.	Lower confidence level (95%)	Upper confidence level (95%)	*p*-value
Intercept		1	8.09	0.10652	5,764.911	7.88	8.30	0.000000
Weight		3	0.00	0.00000	377.657	0.00	0.00	0.000000
Month	1	4	−0.24	0.05549	18.114	−0.34	−0.13	0.000021
Month	2	5	0.01	0.04685	0.017	−0.09	0.10	0.894913
Month	3	6	−0.06	0.04677	1.606	−0.15	0.03	0.205111
Month	4	7	−0.38	0.06983	29.216	−0.51	−0.24	0.000000
Month	5	8	−0.33	0.06063	29.196	−0.45	−0.21	0.000000
Month	6	9	−0.32	0.06157	26.281	−0.44	−0.19	0.000000
Month	7	10	−0.07	0.06588	1.046	−0.20	0.06	0.306321
Month	8	11	0.04	0.05557	0.487	−0.07	0.15	0.485170
Month	9	12	0.15	0.05474	7.091	0.04	0.25	0.007749
Month	10	13	0.39	0.04364	78.479	0.30	0.47	0.000000
Month	11	14	0.44	0.04001	119.440	0.36	0.52	0.000000
Incoterms®	C/D	15	−0.78	0.04728	272.654	−0.87	−0.69	0.000000
Scale			12,970.62	65.55335		12,842.77	13,099.74	

**Table 6:** Estimation model for margin loss in export operations for Company X in 2021.

Effect	Parameter estimates Distribution: NORMAL Link function: LOG							
	Level of effect	Column	Estimate	Standard error	Wald stat.	Lower confidence level (95%)	Upper confidence level (95%)	*p*-value
Intercept		1	8.119	0.04140	38,460.67	8.038	8.200	0.000000
Weight		2	−0.000	0.00001	12.32	−0.000	−0.000	0.000449
Distance		3	0.000	0.00000	207.28	0.000	0.000	0.000000
Month	1	4	−0.144	0.05889	6.01	−0.260	−0.029	0.014262
Month	2	5	0.027	0.04929	0.31	−0.069	0.124	0.578675
Month	3	6	0.021	0.04663	0.20	−0.070	0.112	0.651081
Month	4	7	0.009	0.04847	0.03	−0.086	0.104	0.854085
Month	5	8	0.026	0.04583	0.32	−0.064	0.116	0.572769
Month	6	9	0.103	0.04447	5.41	0.016	0.191	0.020020
Month	7	10	−0.172	0.07530	5.20	−0.319	−0.024	0.022580
Month	8	11	−0.043	0.06631	0.42	−0.173	0.087	0.516577
Month	9	12	−0.117	0.07427	2.46	−0.262	0.029	0.116664
Month	10	13	0.096	0.06342	2.30	−0.028	0.221	0.129300
Month	11	14	0.028	0.06407	0.19	−0.097	0.154	0.660445
Incoterms®	C/D	15	0.234	0.02231	110.46	0.191	0.278	0.000000
Scale			5,441.511	39.51849		5,364.605	5,519.520	

In the estimation model for margin loss in import operations for Company X, the findings reveal a notable decrease in the margin per operation by −0.78 for transactions conducted under Incoterms® within the grouping C/D, which do not utilize transport remit, compared to those within the grouping E/F, *ceteris paribus* (c.p.), holding other factors constant. The control variables, with the exclusion of distance, demonstrate statistical significance, indicating that the most favorable margins were typically achieved between September and November, whereas the least favorable were observed from April to June. As for export operations, the margin per operation increases by 0.234 for transactions executed with Incoterms® grouping C/D compared to those that did not within grouping E/F, c.p. Similarly, all control variables—weight, distance, and month—were statistically significant.

Regarding the estimation of margin loss in import and export operations per 1 kilogram, the analysis delves into transactions executed under different Incoterms® groupings to demonstrate significant variations. In import operations, transactions conducted under Incoterms® within grouping C/D exhibit a notable decrease by −1.746 compared to those executed in grouping E/F, c.p. (Table 7). Conversely, in export operations, the margin per 1 kilogram per operation increases by 0.040 for transactions executed under Incoterms® grouping C/D compared to those executed in grouping E/F, c.p. (Table 8). These results highlight how the Incoterms® groupings significantly impact the financial results of both import and export operations, providing insight into the complexities of the Polish transportation and logistics sector. Correspondingly, the statistical significance of the control variables—weight, distance, and month—in both models reinforces the robustness of the analysis, providing valuable insights for informed decision-making and strategic planning in optimizing operational efficiency and profitability in Company X’s international trade activities.

**Table 7:** Estimation model for margin loss in import operations based on performance per 1 kilogram for Company X in 2021.

Effect	Parameter estimates Distribution: NORMAL Link function: LOG							
	Level of effect	Column	Estimate	Standard error	Wald stat.	Lower confidence level (95%)	Upper confidence level (95%)	*p*-value
Intercept		1	13.637	0.13073	10,881.98	13.381	13.894	0.000000
Weight		2	0.000	0.00002	462.69	0.000	0.000	0.000000
Distance		3	−203.542	4.29997	2,240.67	−211.970	−195.114	0.000000
Month	1	4	−1.364	0.25454	28.74	−1.863	−0.866	0.000000
Month	2	5	−1.670	0.08011	434.47	−1.827	−1.513	0.000000
Month	3	6	−0.047	0.09772	0.23	−0.239	0.145	0.630509
Month	4	7	−2.170	0.10519	425.77	−2.377	−1.964	0.000000
Month	5	8	0.908	0.11955	57.64	0.673	1.142	0.000000
Month	6	9	−5.063	0.12853	1,551.42	−5.314	−4.811	0.000000
Month	7	10	5.540	0.22138	626.27	5.106	5.974	0.000000
Month	8	11	−1.569	0.12494	157.60	−1.813	−1.324	0.000000
Month	9	12	2.150	0.13836	241.56	1.879	2.422	0.000000
Month	10	13	3.273	0.17731	340.84	2.926	3.621	0.000000
Month	11	14	1.407	0.25072	31.51	0.916	1.899	0.000000
Incoterms®	C/D	15	−1.746	0.06921	636.41	−1.882	−1.610	0.000000
Scale			2,366.161	15.27032		2,336.420	2,396.280	

**Table 8:** Estimation model for margin loss in export operations based on performance per 1 kilogram for Company X in 2021.

Effect	Parameter estimates Distribution: NORMAL Link function: LOG							
	Level of effect	Column	Estimate	Standard error	Wald stat.	Lower confidence level (95%)	Upper confidence level (95%)	*p*-value
Intercept		1	10.352	0.04164	61,802.70	10.270	10.433	0.000000
Weight		2	−0.000	0.00000	36.68	−0.000	−0.000	0.000000
Distance		3	−8.477	0.26893	993.56	−9.004	−7.950	0.000000
Month	1	4	0.285	0.06915	17.01	0.150	0.421	0.000037
Month	2	5	0.021	0.03845	0.31	−0.054	0.097	0.579433
Month	3	6	0.412	0.03417	145.27	0.345	0.479	0.000000
Month	4	7	−0.639	0.08084	62.49	−0.797	−0.481	0.000000
Month	5	8	−0.064	0.05260	1.48	−0.167	0.039	0.223630
Month	6	9	0.072	0.04321	2.77	−0.013	0.157	0.096170
Month	7	10	−0.107	0.08254	1.69	−0.269	0.054	0.193144
Month	8	11	−0.335	0.14141	5.62	−0.612	−0.058	0.017803
Month	9	12	−0.228	0.07124	10.24	−0.368	−0.088	0.001374
Month	10	13	0.179	0.05114	12.28	0.079	0.279	0.000458
Month	11	14	0.402	0.03933	104.25	0.325	0.479	0.000000
Incoterms®	C/D	15	0.040	0.02346	2.95	−0.006	0.086	0.085994
Scale			1,672.408	25.38033		1,623.396	1,722.900	

#### 4.4.2. Assessment of economic impact of import and export operations in 2019 and 2021 for Company X.

Based on the estimation models for assessing margin loss in import and export operations for Company X, standardized economy-wide data indicates a general recovery to pre-pandemic levels in terms of Incoterms® services. Regarding economic data for imports, Company X witnessed an increase in its total forwarding Incoterms® operations from USD 208,021,195,417 in 2019 to USD 271,607,436,786 in 2021, resulting in a net gain of USD 63,586,241,369. Upon closer examination, the Incoterms® formulas that experienced a net gain totaled USD 64,013,769,852, while those with a net loss amounted to USD 427,528,483. Notably, a total of 338,742 import transactions were documented, with roughly 56% falling under the Incoterms® grouping C/D (Table 9). Based on the models evaluating margin loss in import operations, the weighted margin deviation ratios for transactions in grouping E/F were determined to be USD 1,446,572 (+/- USD 48.00). Factoring in Company X’s average profitability, with a return on sales (ROS) of 30.16%, and the industry average profitability of USD 55.00 per TEU, the estimated margin per transaction within the C/D grouping was USD 1,921.87. Adjusting for the deviation factor, the average margin per transaction could reach USD 3,368.44. Extrapolating this to the entire economy implies an average loss of USD 643,783,737 within a confidence interval ranging from USD 634,578,188 to USD 652,989,287.

**Table 9:** Economic data for Incoterms® imports in 2019 and 2021 for Company X, USD.

Incoterms® formula^†^	2019	2021	Difference
CFR	1,766,207,185	2,382,430,285	616,223,099
CIF	11,535,224,291	14,545,984,021	3,010,759,730
CIP	24,559,357,588	29,980,718,984	5,421,361,396
CPT	13,755,421,420	15,906,722,118	2,151,300,699
DAF	309,756,666	150,765,502	(−158,991,164)
DAP	41,446,518,808	60,119,265,184	18,672,746,376
DAT	585,743,469	536,004,403	(−49,739,066)
DDP	21,734,498,018	30,207,037,256	8,472,539,238
DDU	399,340,945	185,976,505	(−213,364,440)
DEQ	2,044,850	11	(−2,044,839)
DES	3,392,307	3,333	(−3,388,974)
DPU	—	499,536,674	499,536,674
EXW	27,527,945,080	32,441,433,880	4,913,488,800
FAS	96,260,394	136,009,532	39,749,138
FCA	22,964,330,423	31,326,220,816	8,361,890,393
FOB	16,438,655,118	21,514,944,598	5,076,289,480
(blank)	24,896,498,856	31,674,383,684	6,777,884,828
Total	208,021,195,417	271,607,436,786	63,586,241,369
C/D grouping	0.558104205	0.568888618	
N = 338,742			

† Delivered At Frontier (DAF), Delivered Duty Paid (DDP), Delivered Duty Unpaid (DDU), Delivered Ex Quay (DEQ), Delivered Ex Ship (DES).

For port turnover, representing 20% of Polish imports by weight, this translates to an annual revenue loss of approximately USD 128 million due to foreign outsourcing of transport remit services. Considering the Port of Gdansk’s significant share of total transshipments at around 46% in 2021, the estimated loss in revenue for Polish forwarding enterprises amounts to USD 84 million. Consequently, there are real losses for the national budget, including USD 13.4 million from VAT and USD 11.3 million from corporate income tax (CIT) and personal income tax (PIT), totaling over USD 24.8 million annually. It is important to note that these losses stem solely from lost margins.

As such, the aspect that cannot be precisely measured is the revenue loss incurred by subcontractors engaged by Polish forwarding enterprises for domestic import handling when Polish importers opt out of transport remit. The estimated revenue loss for Polish partners failing to secure orders from forwarders in import relations totals around USD 177.6 million, while for the national budget, the forfeited revenue from VAT, CIT, and PIT sums up to about USD 74.4 million.

In terms of export transactions for Company X, there was a significant uptick in the total forwarding operations of Incoterms® from USD 223,330,999,676 in 2019 to USD 285,530,032,707 in 2021, resulting in a net gain of USD 62,199,033,031. Delving deeper, Incoterms® formulas experiencing a net gain amounted to USD 62,475,754,560, while those encountering a net loss stood at USD 276,721,529. Export records tallied 150,119 transactions, with approximately 48% conducted under the Incoterms® grouping C/D, signifying operations with transport remit (Table 10). According to the margin loss assessment models for export operations, the weighted margin deviation ratios for grouping E/F transactions, i.e., excluding transport remit, were determined at USD −350,475.93 (+/- USD 29.50). Factoring in the average profitability of Company X’s ROS at 40.01% and the industry average of USD 55.00 per TEU, each transaction within grouping E/F reflected a margin of USD 5,633.78. Adjusting for the deviation factor, the average margin per transaction was recalibrated to USD 5,984.26. This translates to an average economic loss of USD 1,143,724,016, falling within the confidence interval of USD 1,138,078,969 to USD 1,149,369,063 when extrapolated to the economy as a whole.

**Table 10:** Economic data for Incoterms® exports in 2019 and 2021 for Company X, USD.

Incoterms® formula	2019	2021	Difference
CFR	2,027,434,520	2,280,553,640	253,119,120
CIF	8,040,254,400	8,387,672,800	347,418,400
CIP	19,232,090,000	24,071,132,000	4,839,042,000
CPT	12,333,353,200	15,906,799,600	3,573,446,400
DAF	9,315,346	3,595,070	(−5,720,276)
DAP	44,916,236,000	61,294,532,000	16,378,296,000
DAT	843,730,400	699,799,920	(−143,930,480)
DDP	16,874,608,000	26,304,536,000	9,429,928,000
DDU	125,070,624	66,009,496	(−59,061,128)
DEQ	439,904	11,787	(−428,117)
DES	448,749	117,497	(−331,252)
DPU	—	344,936,840	344,936,840
EXW	39,953,116,000	42,186,520,000	2,233,404,000
FAS	123,333,532	56,083,256	(−67,250,276)
FCA	43,923,612,000	59,061,128,000	15,137,516,000
FOB	2,171,365,000	2,928,240,800	756,875,800
(blank)	32,756,592,000	41,938,364,000	9,181,772,000
Total	223,330,999,676	285,530,032,707	62,199,033,031
C/D grouping	0.467804000	0.488036000	
N = 150,119			

For maritime transport, comprising 15% of Polish exports by weight, the loss in revenue amounts to approximately USD 171.5 million annually due to the relinquishment of transport remit services. Notably, the Port of Gdansk, with a 46% share of total Polish forwarding operations in 2021, implies lost revenue of USD 78.9 million for Polish forwarding enterprises. Consequently, there are tangible losses for the national budget, including approximately USD 18.1 million from VAT and USD 14.9 million from CIT and PIT, totaling over USD 33.6 million annually in tax losses solely from export relations. Note, these figures reflect only the losses resulting from forfeited margins for cargo forwarding through the Port of Gdansk.

Similarly, in the context of export operations, the precise revenue loss for subcontractors remains uncertain. Assuming an average margin ranging from 5% to 15% concerning total transport costs, with one-third of these costs allocated to transportation to a Polish port, the estimated loss for Polish enterprises not served amounts to USD 236.7 million. This equates to forfeited national budget revenue from VAT, CIT, and PIT standing at approximately PLN 100.8 million. Overall, to uphold the current transport remit practices would result in hypothetical losses for the Polish economy, totaling USD 1.79 billion in lost revenues for transport remit services and USD 751.9 million in lost budget revenues. This comprises USD 410.9 million attributed to VAT and USD 339.5 million allocated to CIT and PIT, resulting in an estimated cumulative loss of USD 2.54 billion to the Polish economy.

### 4.5. Industry-wide survey

**Fig 2:**
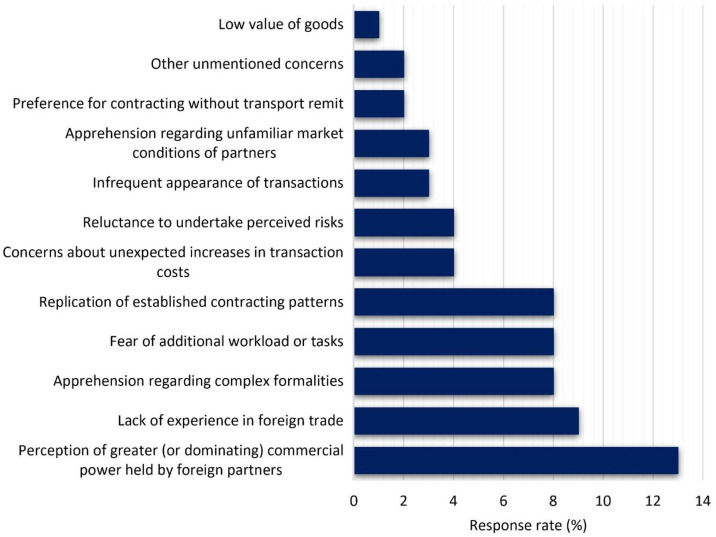
Reasons for Polish enterprises not to utilize transport remit services to their foreign partners in 2021.

The industry-wide survey findings from Polish TSL managers not only validated the results obtained through the quantitative analysis based on government data and the case research of Company X but also provided valuable qualitative insights. Surprisingly, only a negligible 4% of respondents claimed to be entirely unaware of the phenomenon of relinquishing transport remit among their clients. A primary reason identified for Polish enterprises not utilizing transport remit services was the perceived dominance in bargaining power wielded by foreign partners, compelling compliance with their terms (Fig 2). Additionally, respondents highlighted several other contributing factors, including the lack of experience in conducting foreign commercial transactions, the intricate formalities associated with assuming transport remit responsibilities, concerns about increased workload, and a tendency to adhere to past practices of avoiding transport remit. These insights underscore the multifaceted nature of the challenges faced by Polish enterprises in navigating foreign trade dynamics and underscores the need for comprehensive strategies to address these concerns.

## 5. Conclusion

The examination of Polish forwarding enterprises presents a compelling narrative of both challenges and opportunities within the transport remit landscape. Using Company X’s operations as a case example, we uncover a notable inclination toward foreign partners assuming transport remit responsibilities, signaling a trend that warrants closer scrutiny and strategic intervention. Despite facing margin setbacks attributed to the COVID-19 pandemic, the imperative to grasp long-term trends and regional disparities looms large, underlining the strategic significance of informed decision-making to fortify Poland’s competitive edge in global trade. A detailed statistical analysis of Company X’s transport operations highlights the prevalent reliance on transport remit services, with a significant proportion of operations involving Polish partners. Noteworthy trends emerge in import and export transactions, with transport remit slightly more prevalent in exports. The dominance of specific Incoterms® formulas further underscores the complexity and nuances inherent in transport remit practices, with distinct preferences observed across various transaction types.

Moreover, the econometric model developed to assess the impact of transport remit practices unveils far-reaching implications, extending beyond individual companies to affect national budget revenues and subcontractors. The economic ramifications of relinquishing transport remit services, either entirely or by overly utilizing foreign partners, underscore the imperative for strategic alignment and proactive measures to mitigate losses and capitalize on untapped potential. Addressing the multifaceted challenges hindering the expansion of transport remit services among Polish importers and exporters necessitates a comprehensive approach. Efforts encompassing further research, face-to-face expert interviews, and the development of clear guidelines tailored for Polish enterprises are essential to bridge knowledge gaps, streamline processes, and foster wider adoption of transport remit services in the country. Furthermore, initiatives aimed at enhancing education, refining formal and legal systems, and mitigating costs and risks are pivotal to incentivizing Polish operators and nurturing a conducive environment for growth and development in the transport remit market.

Company X’s proactive approach exemplifies how strategic interventions can drive meaningful change. By identifying and leveraging the business benefits of managing transport responsibilities domestically, the company has begun to shift client behavior away from default reliance on foreign partners. This initiative sets a foundation for broader, long-term growth and increased competitiveness in Poland’s logistics sector. Looking ahead, while challenges remain, the potential for progress is substantial. With coordinated efforts from government, industry, and education providers, Polish enterprises can strengthen their role in global trade. Through continued innovation, strategic planning, and capacity building, Poland is well-positioned to unlock the full value of its transport remit capabilities and establish itself as a leading logistics hub in international commerce.
