# Correction: Optimizing international trade: Strategies for retaining or taking over transport remit in Poland’s import and export landscape—A case study of Company X

**DOI:** 10.1371/journal.pone.0341239

**Published:** 2026-01-20

**Authors:** Joanna Bednarz, Monika Grottel, Anna Sperska, Giuseppe T. Cirella, Michał Suchanek, Aneta Oniszczuk-Jastrząbek, Ernest Czermanski

The fifth author’s name is spelled incorrectly. The correct name is: Michał Suchanek. The correct citation is: Bednarz J, Grottel M, Sperska A, Cirella GT, Suchanek M, Oniszczuk-Jastrząbek A, et al. (2025) Optimizing international trade: Strategies for retaining or taking over transport remit in Poland’s import and export landscape—A case study of Company X. PLoS One 20(9): e0332126. https://doi.org/10.1371/journal.pone.0332126
